# Strengthening Social Capital to Address Isolation and Loneliness in Long-term Care Facilities During the COVID-19 Pandemic: Protocol for a Systematic Review of Research on Information and Communication Technologies

**DOI:** 10.2196/36269

**Published:** 2022-03-24

**Authors:** Idrissa Beogo, Drissa Sia, Eric Tchouaket Nguemeleu, Junqiang Zhao, Marie-Pierre Gagnon, Josephine Etowa

**Affiliations:** 1 School of Nursing Faculty of Health Sciences University of Ottawa Ottawa, ON Canada; 2 College of Nursing Rady Faculty of Health Sciences University of Manitoba Winnipeg, MB Canada; 3 Département des sciences infirmières Université du Québec en Outaouais Saint-Jerôme, QC Canada; 4 Département de médecine sociale et préventive École de santé publique Université de Montréal Montréal, QC Canada; 5 Département de gestion d'évaluation et de politique de santé École de santé publique, Université de Montréal Montréal, QC Canada; 6 Faculté des sciences infirmières Université Laval Québec, QC Canada

**Keywords:** ICT, long-term care facilities, COVID-19, social isolation, loneliness, pandemic, implementation sciences, protocol, nursing home, long-term care, mental health, aging, older adults, virtual communication, virtual care, information technology, healthcare, healthcare sector, health care

## Abstract

**Background:**

The COVID-19 pandemic has had the greatest impact in long-term care facilities (LTCFs) by disproportionately harming older adults and heightening social isolation and loneliness (SIL). Living in close quarters with others and in need of around-the-clock assistance, interactions with older adults, which were previously in person, have been replaced by virtual chatting using information and communication technologies (ICTs). ICT applications such as FaceTime, Zoom, and Microsoft Teams video chatting have been overwhelmingly used by families to maintain residents’ social capital and subsequently reduce their SIL.

**Objective:**

Because of the lack of substantive knowledge on this ever-increasing form of social communication, this systematic review intends to synthesize the effects of ICT interventions to address SIL among residents in LTCFs during the COVID-19 period.

**Methods:**

We will include studies published in Chinese, English, and French from December 2019 onwards. Beyond the traditional search strategy approach, 4 of the 12 electronic databases to be queried will be in Chinese. We will include quantitative and intervention studies as well as qualitative and mixed methods designs. Using a 2-person approach, the principal investigator and one author will blindly screen eligible articles, extract data, and assess risk of bias. In order to improve the first round of screening, a pilot-tested algorithm will be used. Disagreements will be resolved through discussion with a third author. Results will be presented as structured summaries of the included studies. We plan to conduct a meta-analysis if sufficient data are available.

**Results:**

A total of 1803 articles have been retrieved to date. Queries of the Chinese databases are ongoing. The systematic review and subsequent manuscript will be completed by the fall of 2022.

**Conclusions:**

ICT applications have become a promising avenue to reduce SIL by providing a way to maintain communication between LTCF residents and their families and will certainly remain in the post–COVID-19 period. This review will investigate and describe context-pertinent and high-quality programs and initiatives to inform, at the macro level, policy makers and researchers, frontline managers, and families. These methods will remain relevant in the post–COVID-19 era.

**International Registered Report Identifier (IRRID):**

DERR1-10.2196/36269

## Introduction

### Background

The COVID-19 pandemic has disproportionately harmed older adults and subsequently heightened their social isolation and loneliness (SIL) [1,2] and suicidal vulnerability [3-5]. Long-term care facilities (LTCFs) are the most profoundly affected sector with the highest mortality rate of 81% [6,7]. Apart from being the hardest hit during the pandemic, residents have already been experiencing long-lasting SIL before the beginning of the COVID-19 pandemic [8]. To address this SIL, information and communication technologies (ICTs) have become key resources for families to maintain a socio-emotional connection with their loves secluded in LTCFs [9]. Families remain LTCF residents’ strongest support system. The Canadian Institutes for Health Information contends that the majority (82%) of older adults need family involvement in their instrumental activities of daily living [10-12].

Systematic [13] and Cochrane [14] reviews undertaken before the COVID-19 pandemic were inconclusive with regard to the effect of ICT in reducing SIL in older adults. However, the pandemic has confirmed the importance of ICT’s applications worldwide to provide remote chatting conferencing—a feature that maintains vital interactions between families and their loved ones. Owing to stringent public health restrictions on physical access to LTCFs, ICT has been the only alternative to maintaining social capital and subsequently reducing SIL among seniors in LTCFs [15]. Residents in these settings are more fraught with SIL, varying from 40% [16] to 71.6% [17]. As noted by Abbasi [18], during the current COVID-19 pandemic, SIL resulted in an uptake of antidepressants, antipsychotics, and anxiolytics in LTCF residents. Along with being physically and psychologically separated from the community, many older adults find that relocation to a LTCF itself is a stressful life event. The literature clearly contends that the majority of older adults are reluctant to be relocated [19-21]. Many older adults are widowed, under guardianship or tutorship, and an unknown proportion—4% in the Canadian general population in 2018 [22]—sel-identify as lesbian, gay, bisexual, transgender, questioning, or two-spirit [23]. These vulnerable older adults often become further isolated once admitted into a LTCF and experience greater levels of SIL [24], which underlines the necessity for providing interactive connections with others.

SIL negatively affects quality of life and is associated with an increase in all-cause mortality, an effect that is slightly stronger in men than in women [25-27]. Owing to the public health restrictions imposed during the COVID-19 pandemic, LTCF residents had limited contact with staff and were often secluded in their rooms and no longer able to partake in communal meals, in-person activities, or family visits. The threat of infection and the loss of contact with loved ones has contributed to SIL and has likely exaggerated subsequent negative outcomes in older people. Nevertheless, SIL in LTCFs has just started being considered in the literature. With an increasing incidence of COVID-19, the use of ICT applications has skyrocketed to facilitate social communication [15], providing a necessary support for older people in long-term care [28].

The use of ICT to reduce SIL has been extensively studied, including the effects of internet-based interventions [29-33] and Humanoid Robot approaches [34-36]. Methods currently used in LTCFs to connect older adults with their families and friends range from the conventional telephone to web-based platforms such as Skype, FaceTime, Zoom, or Google Meet (for a review see Banskota et al [37] or Chen and Schulz [38]). Zamir et al [39] employed an intercare home group “Skype” to reduce SIL in 3 care homes and found that video calls reduced feelings of loneliness in residents seemed acceptable and was a feasible, low-cost model, especially during times of public crisis such as during the COVID-19 pandemic.

### Research Question and Objectives

This review intends to assess the effect of ICT interventions implemented in LTCFs to address SIL among residents during the COVID-19 pandemic. The following objectives will be considered to address this research question:

1. To synthesize the effects of ICT interventions to address SIL in LTCF residents during the COVID-19 period;
2. To identify studies that use ICT, namely through a varied function of communication such as messaging or chat, video, voice mail, or photo as a strategy for interaction and connection with older family members living in LTCFs;
3. To measure the impact of ICT on the interaction between families and their family members in LTCF facilities.

### Rationale for This Study

Older adults have been relying on family members to monitor their “health, well-being, and safety” through virtual visits during the COVID-19 pandemic [40]. SIL in older people has been identiﬁed as a risk factor for premature mortality [27] for both poor physical (eg, cardiovascular and obesity) [41-43] and psychiatric health (eg, depression and anxiety) [44]. The significance of communication technology has been featured prominently in local and national news outlets that have highlighted the stories of residents and families being connected via various ICT applications during the pandemic [45]. Liotta et al [46] explained how increased social connectedness was a powerful tool for nursing home residents that it decreased SIL during the pandemic. Thus, innovative use of digital tools can provide a method to address this urgent public health matter on a long-term basis [47]. As opportunities offered by ICT applications have the potential for long-term solutions for the COVID-19 pandemic and postpandemic period, this systematic review will help inform policy and practice interventions in this area.

The proposed systematic review is also necessary to shed light on the pre–COVID-19 pandemic literature on the impact of ICT on SIL. Systematically examining the evidence on the association between ICT and SIL will help establish up-to-date knowledge to develop best practices and support evidence-based policy decision-making.

## Methods

### Identification of Data Sources and Studies

This systematic review will be conducted following the Cochrane Collaboration methods [48] and the Preferred Reporting Items for Systematic Review and Meta-Analysis Protocols (PRISMA-P) checklist to ensure the completeness of this protocol. We will consult the Synthesis Without Meta-analysis (SWiM) guidelines to guide the use of alternative synthesis methods. The review is registered with the OSF registries [49]. The search will be performed in English, French, and Chinese. A pilot exploratory search on Ovid MEDLINE will be undertaken to create a robust string that is well calibrated in order to improve the likelihood of retrieving articles that are as relevant as possible. This interactive process will include both free vocabulary and descriptors. Beyond the traditional approach that emphasizes on French or English search strings, this review will be enhanced by searching on Chinese databases. Tables 1 and 2 exhibit the search strategies in English for the Ovid MEDLINE database and in Chinese for the China National Knowledge Infrastructure (CNKI) database. The final search strategy involved the following databases: PsycINFO, Ovid MEDLINE, Embase, CINAHL, Cochrane Library, Web of Science, Communication & Mass Media Complete, Association for Computing Machinery (ACM) Digital Library, IEEE Xplore, CNKI, WanFang, Weipu (VIP), and SinoMed. Two review authors, the principal investigator (IB) and a PhD student (JZ), who is a native Mandarin speaker and writer, will canvas all the titles and abstracts; a third review author (DS) will resolve the conflicts. A pilot test will be implemented using a pilot-tested algorithm, which is shown in Figure S1 in Multimedia Appendix 1.

**Table 1:** Ovid MEDLINE search strategy (to be modified as needed for other databases).

Number	Query
#1	(lonel* or 'social connect*' or connectedness or 'social distanc*' or aloneness or solitude or Seclu* or confin* or separat* or quarantine* or remote* or 'emotional isolation' OR Quarantine). .ab,kw,ti.
#2	exp Loneliness/ or exp Quarantine/
#3	#1 OR #2
#4	(isolat* or deprivation or network or support).ab,kw,ti. . AND social.ab,kw,ti.
#5	exp Social Isolation/
#6	#4 OR #5
#7	#3 OR #6
#8	('Long-Term Care' or 'Assisted-Living Facilit*' or 'Homes for the Aged' or 'Nursing Home*' or Geriatrics or 'homes for the aged' or 'Housing for the Elderly').ab,ti.
#9	(long-term-care OR Geriatrics OR 'Older Adult*' OR elde* OR senior OR aged OR retirement).ab,kw,ti. AND (Home, OR facilit* OR residen*).ab,kw,ti.
#10	exp Nursing Homes/ OR exp Housing for the Elderly/
#11	#8 OR #9 OR #10
#12	(Pandemi* or epidemi* or andemi* OR Outbreak) or (coronavirus or COVID-19 or SARS-COV2 ).ab,ti.+kw
#13	exp pandemic/ or exp pandemic/ OR COVID-19/ OR SARS-CoV-2/
#14	#12 OR #13
#15	('Digital technology' OR Zoom OR facebook OR 'information technology' OR Skype OR 'FaceTime' OR 'cell phone*' or smartphone* or on-line or online or web-based or 'webbased' or 'web based' or 'world wide web' OR 'Cellular Phone*' or 'mobile phone*' or internet).ab,kw,ti.
#16	information technology/ OR information technology/ OR Cell Phone/ OR smartphone/ OR Online Social Networking/ OR internet/
#17	#15 OR #16
#18	#7 AND #11 AND #14 AND #17

**Table 2:** Planned search strategy in CNKI.

Number	Query
#1	TKA=社会资本 OR 社会隔绝 OR 社会隔离 OR 社会疏远 OR 社会疏离 OR 社会距离 OR 社群隔离 OR 隔离 OR 空间隔离 OR 情感隔离 OR 孤独 OR 孤独感 OR 生活质量
#2	TKA=老年
#3	TKA=传染病 OR 疫情 OR 冠状病毒 OR COVID-19 OR 新型冠状病毒肺炎 OR 新冠肺炎 OR 冠状病毒肺炎
#4	TKA=计算机通信网络 OR 信息通信技术 OR 信息通讯技术 OR 信息技术 OR 网络 OR 互联网 OR 智能手机 OR 便携式电话 OR 手机 OR 电话 OR 短信 OR 视频 OR 微信
#5	#1 AND #2 AND #3 AND #4

### Selection and Data Extraction

All retrieved articles will be uploaded into Rayyan Intelligent Systematic Review [50]. After the removal of duplicates, 2 reviewers will independently screen the titles and abstracts identified by the literature search for inclusion. By April 30, 2022, the full text of potentially relevant articles will be screened to determine final inclusion, followed by a data extraction phase. To increase the reliability of screening by the two independent reviewers, a random sample of articles will be screened on the basis of the eligibility criteria in a pilot test phase. For the screening, a structured algorithm that was previously validated by IB, SD, and ETN will be used.

A standardized data extraction grid that has been developed and piloted will be employed to extract data from all full texts. This includes the following: authors, year of publication, language, design, objectives, participants’ characteristics, ICT intervention, outcomes, population, setting, and the fields of the AMSTAR checklist. The *K* statistic will then be calculated to determine the intrarater agreement for study inclusion [51]. Studies excluded during the screening phase will be recorded along with the reason for exclusion by each reviewer. As we intend to complete the review by October 2022, an updated search will be run shortly before this time in order to capture any recent peer-reviewed publications.

### Quality Assessment

Given the potential heterogeneity in study designs, IB and JZ will independently assess the methodological quality of studies using the design-specific appraisal tool the Cochrane risk-of-bias (RoB 2) for randomized clinical trials, or the Newcastle-Ottawa Scale also used in nonobservational cohort and case-control studies.

### Inclusion and Exclusion Criteria

Inclusion and exclusion criteria will be based on the Population, Interventions, Comparators and designs, Outcomes (PICO) framework, summarized below.

#### Population (P)

This systematic review will consider studies that target SIL reduction as an outcome in older adults aged 65 years and over living in LTCFs (eg, nursing homes or assisted living arrangements). We will exclude studies of the following persons: (1) with a terminal illness, or (2) who are hospitalized, or (3) with severe neurocognitive disorders, or (4) with severely impaired cognition (measured by specific tools such as the Mini-Mental State Examination [52]), or (5) targeting community dwellers.

#### Intervention (I)

This systematic review will focus on the use of information technology, namely through communication modes such as chat, video, voice mail, or photo, which maintain or improve the connection between the older adults in LTCFs and their families. This sector of the Canadian health system—and possibly worldwide—appears to be the poor counterpart of all the segments, above all in the new technologies of communication [53].

In addition to regular communication technology such as the telephone, the main ICT intervention component must be based on the use of the internet to fulfill social networking. This is one of 7 elements that can help older adults maintain their independence, proposed in a white paper by the Center for Technology and Aging [54]. Targeted interventions can be delivered individually or in groups and can take place over one or more sessions of various time frames. Any type of digital tool will be considered, including computers, smartphones, or tablets, with the ultimate goal of addressing SIL using commercial applications including Facebook or Zoom for conversation. Any form of connection involving an important face-to-face component in the conversation, or for the purpose of medical treatments, will be excluded.

#### Comparator (C) or Designs

We will include quantitative studies, specifically randomized controlled trials (RCTs) and quasi‐RCTs (including cluster designs), quasi-experimental, cohort, cross-sectional, and pre-post intervention studies. Qualitative and mixed methods studies will be also included. We will exclude all ICT-based therapeutic interventions, although they also have an interactive component and are capable of reducing SIL. Studies that compare ICT interventions to alternative ones such as visits through widows or contactless control groups during the pandemic will be included. Telehealth or telemedicine, as defined by the World Health Organization [55], although delivered by video, will be excluded as their main intention is not SIL reduction. Further comparison is foreseen through between-group comparisons involving; for instance, phone calls versus calls with a visual component.

#### Outcome (O)

This systematic review will target the following outcomes, irrespective of whether a psychometric measure is used or not.

The primary outcomes were as follows:

- Measures of SIL (ie, scores on any appropriate and validated tool);
- Measures of SIL through proxy outcomes including the following: companionship, friendship, feeling of being forgotten and not belonging, and connection with family.

We will exclude interventions that include an important face-to-face component or technologies that do not support an interactive component.

The secondary outcomes were as follows:

- Self-report measures of symptoms of depression (ie, scores on any self-report questionnaire that is designed to quantify the severity of symptoms of depression);
- Self-report measures of quality of life (ie, scores on any self-report questionnaire that is designed to allow people to rate their quality of life, either overall or within specific domains).

#### Timeline (T)

This study was carried out over the COVID-19 pandemic from December 2019 onward.

### Data Synthesis and Analysis

Extracted data from all included studies will be summarized in tabular format. Data will be categorized and aggregated by type of intervention and type of setting (eg, nursing homes or assisted living arrangements). A narrative synthesis will be completed. Significant as well as nonsignificant results will be collected, analyzed, and discussed within the relevant outcome category. We plan to run a subgroup narrative analysis. A meta-analysis is planned on the basis of the quality and quantity of data, and heterogeneity will be measured through *I*^2^ statistics. All the limitations will be discussed.

### Availability of Data and Material

The data sets generated and analyzed during this study, which would be necessary to interpret, replicate, and build on the findings reported in the review article will be made publicly available as requested by the funding institution. All requests should be addressed to the corresponding author as these data will be stored on a secured server of the Université de Saint-Boniface.

### Ethical Considerations

As this systematic review is part of a “Social isolation and loneliness project,” we received ethical approval from the Ethics Committees for Research of the University of Ottawa (H-08-21-7314) the University of Moncton (dossier 2021-073) and the Research Ethics Board of the Primary Care and Population Health Research Sector of the CIUSSS of the Capitale-Nationale (2021-2303, _SPPL).

### Patient involvement

No patients will be involved. Patients will not be invited to comment on the study protocol design and were not consulted as to how this work may inform patient-relevant outcomes or how a patient might interpret results. However, findings will be disseminated to the public and health care professional networks via conferences, publications, and presentations.

## Results

In this review, no patient will be involved. Data extraction and analysis, as well as writing of the manuscript, are expected to be completed by the end of summer 2022.

## Discussion

According to the International Federation on Ageing, the number one emerging issue faced by seniors in Canada is keeping older people socially connected and active [56]. SIL can be a chronic issue that burdens older adults. This has sharply augmented with the arrival of COVID-19. Active and vigorous programs exist throughout the world to address SIL such as the following: “End Loneliness” in the United Kingdom [57], Danmark spiser sammen [58], “ALONE” of Ireland [59], or even “Better Together,” the Canadian family and caregiver presence initiative [60,61] that has resulted in a significant involvement of families (ie, 20% of families spending over 10 hours per week in supportive activities) [62]. Despite this, more work is needed, and ICT interventions are a good fit.

Virtual communication and technologies that have come to the forefront as the primary mode for LTCF residents during the COVID-19 pandemic appear to be a promising new avenue to maintain social connections and capitalize on the ties among families, their loved ones, and the “outside world.” Furthermore, many older adults have higher levels of eHealth literacy with the baby boomer generation becoming older seniors. Nonetheless, many LTCFs do not have the technological capabilities to support modern-day technologies. This is one area of health care system that the COVID-19 pandemic has shed light on. Indeed, in 2020, Canada Infoway reported that LTCFs health technology is the least funded component of the entire health system; a reality that may also be applicable to the rest of the world [53].

At the same time, the current number of studies on ITC does not reflect the mortality inflicted by COVID-19 in long-term care. When and if visiting condition return to “normal,” greater efforts will be required to further develop and promote a secure way of virtually connecting LTCF residents with their loves in the community. Furthermore, initiatives should be tailored to address individual social isolation needs. This review is a step toward highlighting the need for more high-quality program evaluation of interventions and other initiatives implemented in the course of the COVID-19 pandemic. The findings of this systematic review will help draw attention from relevant stakeholders of health systems, or specifically LTCF-oriented ones, to address this urgent issue. Although SIL is a socially complex issue that requires a multi-sectorial approach, knowledge gathered and synthesized from this exercise may inform the actions of governments, researchers, and frontline LTCF managers.

## Appendix

Multimedia Appendix 1 First round screening algorithm.

## Abbreviations

CIHR: Canadian Institutes for Health Research
CNKI: China National Knowledge Infrastructure
ICT: information and communication technologies
LTCF: long-term care facility
PICO: Population, Interventions, Comparators and designs, Outcomes
PRISMA-P: Preferred Reporting Items for Systematic Reviews and Meta-Analyses Protocols
RCT: randomized controlled trial
SIL: social isolation and loneliness
SWiM: Synthesis Without Meta-analysis
