# Supplementary figures and images for: Strengthening Social Capital to Address Isolation and Loneliness in Long-term Care Facilities During the COVID-19 Pandemic: Protocol for a Systematic Review of Research on Information and Communication Technologies
Source: JMIR Res Protoc. 2022 Mar 24;11(3):e36269. doi: 10.2196/36269 (PMC8955240; doi:10.2196/36269)

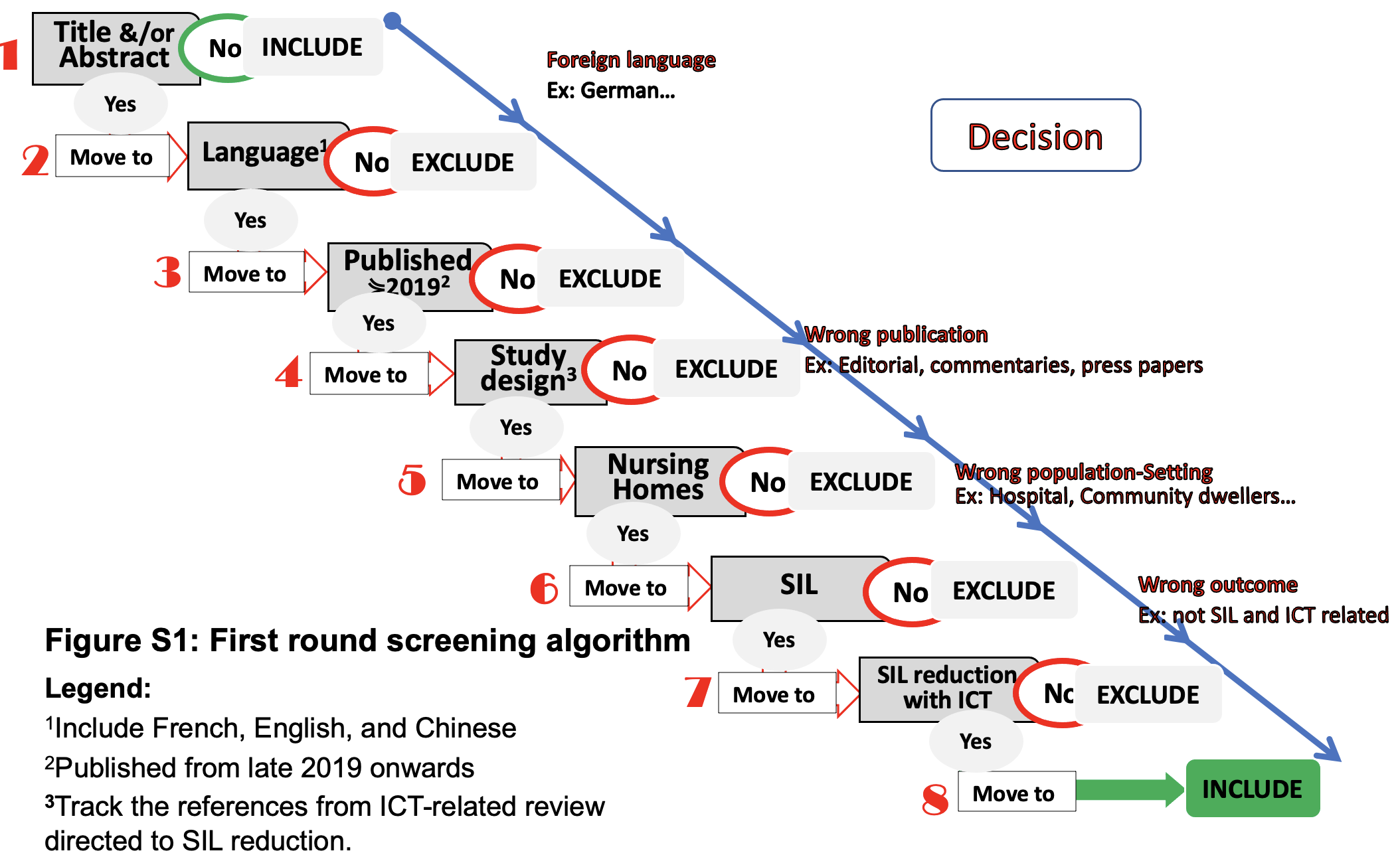

Supplement: Multimedia Appendix 1 [file resprot_v11i3e36269_app1.png]
